# Place de la splénectomie dans la prise en charge de l’hypertension portale non cirrhotique: à propos de 3 cas

**DOI:** 10.11604/pamj.2017.28.84.11712

**Published:** 2017-09-27

**Authors:** Mohamed Said Belhamidi, Salah Eddine Hammi, Mohamed Bouzroud, Mustapha Benmoussa, Abdelmounaim Ait ali, Ahmed Bounaim

**Affiliations:** 1 Service de Chirurgie Viscérale I, Hôpital Militaire d’Instruction Mohamed V, Université de Souissi, Rabat, Maroc; 2 Service de Médecine Interne, Hôpital Militaire d’Instruction Mohamed V, Université de Souissi, Rabat, Maroc

**Keywords:** Hypertension portale, varices oesophagiènnes, hypersplénisme, splénectomie, Portal hypertension, oesophageal varices, hypersplenism, splenectomy

## Abstract

L’hypertension portale non cirrhotique est une affection décrite pour la première fois par Guido BANTI en 1898 comme une affection associant une hypertension portale avec splénomégalie et anémie sur foie sain. Le diagnostic repose sur l’échographie abdominale, la splénoportographie et la biopsie hépatique. Le but de notre travail est d’évaluer la place de la splénectomie dans l’hypertension portale non cirrhotique à travers une étude rétrospective portant sur 3 malades dont 2 femmes et un homme pris en charge dans notre formation entre Janvier 2010 et Septembre 2016. Le diagnostic de l’hypertension portale idiopathique a été basé sur les critères suivants : une hypertension portale, la présence des varices oesophagiènnes avec une splénomégalie, l’absence de cirrhose ou d’autres affections hépatiques responsables de l’hypertension portale. La splénectomie a été réalisée chez les 3 malades. L’évolution après la splénectomie était marquée par la normalisation des signes cliniques, radiologiques et biologiques de cette affection, avec absence de récidive des varices œsophagiennes. La splénectomie associée à la ligature des varices œsophagiennes pourraient être suffisantes pour traiter ce syndrome et surtout ses conséquences sans avoir recours à une dérivation spléno-rénale.

## Abstract (en)

Non-cirrhotic portal hypertension was first described by Guido BANTI in 1898 as a condition characterized by the association of portal hypertension with splenomegaly, anemia and healthy liver. The diagnosis was based on abdominal ultrasound, splenoportography and liver biopsy. Our study aimed to evaluate the role of splenectomy in non-cirrhotic portal hypertension. We conducted a retrospective study of 3 patients (2 women and 1 man) treated by our staff over the period January 2010 -September 2016. The diagnosis of idiopathic portal hypertension was based on the following criteria: portal hypertension, the presence of oesophageal varices associated with splenomegaly, the absence of cirrhosis or of other liver disorders responsible of portal hypertension. All patients underwent splenectomy. Outcome after splenectomy was marked by the standardization of clinical, radiological and biological signs of this disease associated with the absence of oesophageal varices recurrence. Splenectomy associated with ligation of oesophageal varices may be sufficient to treat this syndrome and especially its consequences without using splenorenal bypass.

## Introduction

L’hypertension portale non cirrhotique ou idiopathique ou sclérose hépatoportale, appelée encore syndrome de BANTI, est une affection rare, caractérisée par la présence d’une augmentation de la pression portale secondaire à une sclérose da la paroi des petites branches portales intrahépatiques, avec un foie non cirrhotique [1]. Cette entité a été décrite pour la première fois par Guido BANTI en 1898 comme une affection associant une hypertension portale avec splénomégalie et anémie sur foie sain [2]. Sa pathogénie reste encore mystérieuse. Le syndrome de BANTI survient essentiellement chez les adultes avec prédominance féminine [3]. Le diagnostic repose sur l’échographie abdominale, la splénoportographie et la biopsie hépatique pour éliminer une hépathopathie. La prise en charge thérapeutique vise à juguler les complications de l’hypertension portale en se basant sur des moyens médicaux, instrumentaux et surtout chirurgicaux. Le but de notre travail est d’évaluer la place de la splénectomie dans l’hypertension portale non cirrhotique à travers une série de 3 cas colligés entre 2010 et 2016 au niveau du service de chirurgie viscérale de l’hôpital militaire d’instruction mohamed V.

## Méthodes

Il s’agit d’une étude rétrospective portant sur 3 malades pris en charge dans notre formation entre Janvier 2010 et Septembre 2016. Tous nos malades étaient suivis en médecine interne pour splénomégalie. Les 3 malades ont bénéficiés d’une échographie hépatosplénique et une fibroscopie oeso-gastrique. La biopsie hépatique a été réalisée chez un malade (cas 3). Le diagnostic de l’hypertension portale idiopathique a été basé sur les critères suivants : une hypertension portale définie par un gradient portohépatique de plus de 10 mmHg, la présence d’une splénomégalie, l’absence de cirrhose ou d’autres affections hépatiques responsables de l’hypertension portale. La ligature des varices oesophagiennes sous fibroscopie et une splénectomie ont été réalisées chez les 3 patients. Tous les malades ont reçu, avant la splénectomie, une vaccination antipneumococcique et antimeningococcique. La voie d’abord était une laparotomie sous costale gauche chez 2 malades (cas 1 et 3) et une laparotomie médiane chez le cas 2.

## Résultats

**Figure 1:**
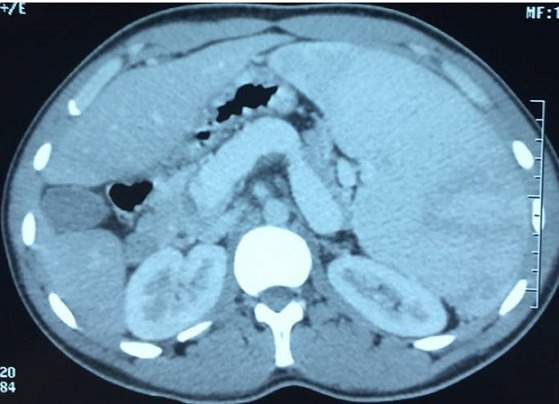
TDM abdominale en coupe axiale objectivant une splénomégalie avec dilatation de la veine splénique et de l’axe spléno-mésaraique

Il s’agit de 3 patients dont 2 femmes et un homme, âgés entre 22 et 41 ans. Ils n’avaient aucun antécédent pathologique notable. 2 malades ont consulté pour des douleurs de l’hypochondre gauche à type de pesanteur et le troisième avait un syndrome anémique. A l’examen clinique, la splénomégalie était la seule anomalie retrouvée chez les trois malades. Il n’y avait pas d’hépatomégalie ni d’ictère ni de matité déclive en faveur d’une ascite ou de circulation veineuse collatérale. Sur le plan radiologique, L’échographie abdominale avec doppler du tronc porte et de la veine splénique étaient en faveur d’une splénomégalie importante avec une hypertension portale chez tous les malades avec une thrombose partielle de la veine porte chez un seul malade. Par ailleurs, il n’y avait pas d’anomalie de la morphologie hépatique ni d’ascite (Tableau 1). L’angio-TDM, réalisée chez un seul malade, a confirmé les données de l’échographie (Figure 1). Chez tous les patients, la fibroscopie œsogastroduodénale a montré des varices œsophagiennes stade 3 sans signes d’hémorragie. Sur le plan biologique, une pancytopénie a été retrouvée chez les 3 cas en faveur d’un hypersplénisme. Par ailleurs, il n’avait ni cytolyse ni de choléstase avec une sérologie hépatitique négative. La biopsie hépatique a objectivé une fibrose portale intrahépatique sans cirrhose.

**Tableau 1:** Résultats du bilan radiologique chez les 3 malades

		Cas 1	Cas 2	Cas 3
échographie	Splénomégalie: SMG	21 cm	27 cm	19 cm
	Diamètre du tronc porte	12 mm	20 mm	19 mm
	Thrombose portale	absente	présente	absente
TMD		Non faite	Non faite	SMG avec importante dilatation du TP avec thrombus endoluminal
IRM		Non faite	SMG avec thrombose portale	Non faite

**Figure 2:**
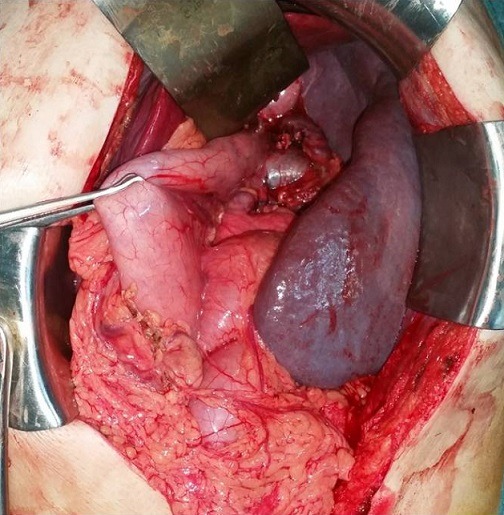
Vue opératoire montrant la splénomégalie avec dilatation de la veine splénique

**Figure 3:**
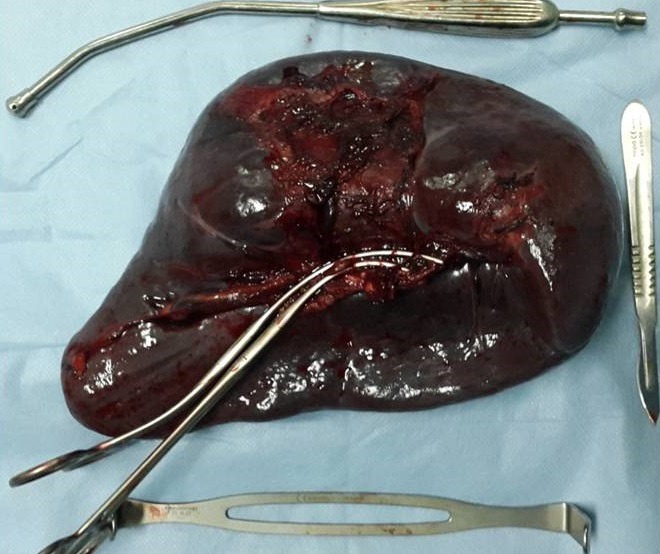
Pièce de splénectomie

**Figure 4:**
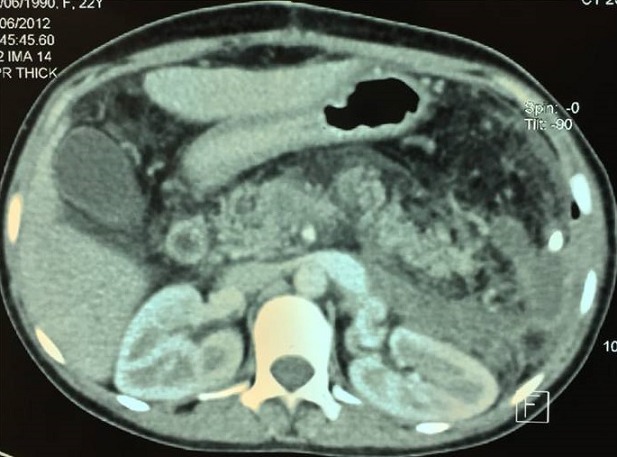
TDM abdominale en coupe axiale montrant une collection de la loge splénique et une thrombose du tronc porte après splénectomie

Les suites opératoires après la splénectomie étaient simples chez 2 malades (Figure 2, Figure 3). Une malade (cas 1) a présenté des douleurs abdominales avec une fièvre chiffrée à 38,5 au 4^ème^ jour postopératoire. La TDM abdominale a montré une collection abcédée dans la loge splénique sans épanchement libre dans la cavité péritonéale avec une thrombose portale (Figure 4). La patiente a bénéficié d’un drainage percutanné échoguidé de la collection et a été mise sous antibiotiques et anticoagulants. Chez tous les cas, l’évolution a été marquée par la normalisation des 3 lignés hématologiques (Tableau 2) avec absence de récidive des varices oesophagiennes.

**Tableau 2:** Numération formule sanguine montrant la résolution de la pancytopénie après splénectomie

	Numération formule sanguine	Cas 1	Cas 2	Cas 3
Avant splénectomie	Hemoglobine (g/dl)	5,3	7,5	9,2
	Globules rouges (10^6^/ mm^3^)	2,5	3,75	3
	Globules blancs (/mm^3^)	1100	1200	1500
	Plaquettes (/mm^3^)	32000	81000	35000
Après splénectomie (J+2)	Hemoglobine (g/dl)	10,3	11	11,7
	Globules rouges (10^6^/ mm^3^)	4,2	5	4,7
	Globules blancs (/mm^3^)	6000	4500	7000
	Plaquettes (/mm^3^)	110000	144000	130000

## Discussion

L’hypertension portale non cirrhotique (HTPNC) est une entité rare, pouvant toucher tous les pays du monde, mais elle est de loin plus fréquente dans les pays en voie de développement avec un niveau socio-économique moins favorable comme l’Inde dont la fréquence de la maladie st estimée à environ 30% des hypertensions portales [4]. Elle touche essentiellement l’adulte jeune entre la 3^ème^ et la 4^ème^ décennie [5]. L’âge moyen de nos malades était de 31,5 ans avec un niveau socio-sanitaire défavorable. Jusqu’à présent, aucun facteur étiologique précis n’a été identifié [6, 7], malgré que dans certaines séries, un facteur prothrombique général, acquis ou héréditaire a pu être détecté chez près de ma moitié des malades [8]. L’HTPNC est caractérisée par la présence d’une hypertension portale sur un foie sain avec des varices gastro-œsophagiennes et une splénomégalie avec souvent un hypersplénisme. Plus de la moitié des malades peuvent être asymptomatiques au moment du diagnostic qui est alors fait lors des investigations des anomalies biologiques ou radiologiques de découverte fortuite [9]. Nos 3 malades étaient asymptomatiques mise à part une pâleur cutanéo-muqueuse chez une patiente.

L’échographie abdominale objective souvent une dilatation de l’axe porto-systémique associée un épaississement des parois de la veine porte et de ses branches principales avec une splénomégalie sans anomalies de la morphologie hépatique [10]. L’écho-doppler est utile pour identifier une thrombose des branches intrahépatiques de la veine porte [11, 12]. Les résultats de l’échographie et du doppler chez nos patients étaient identiques à ceux de la littérature. La fibroscopie oeso-gastro-duodénale objective des varices œsophagiennes chez 85 à 95% des malades [13, 14]. Tous nos malades avaient des varices œsophagiennes sans signes d’hémorragie.

Sur le plan histologique, il existe souvent une fibrose dense sur la paroi de la veine porte ainsi qu’une oblitération de ses petites branche [15-17]. La biopsie hépatique réalisée chez l’un de nos patients, a objectivé une fibrose portale intrahépatique sans signes de cirrhose. Sur le plan biologique, la fonction hépatique est généralement normale. Une pancytopénie est souvent présente et secondaire à un hypersplénisme. Il est beaucoup plus fréquent dans l’HTPNC que dans l’hypertension portale sur cirrhose. Sa fréquence varie de 22 à 88%. La sévérité de l’hypersplénisme est en rapport avec la gravité de la splénomégalie [18, 19].

La prise en charge de ce syndrome est basée sur un ensemble de moyens médicaux, instrumentaux et chirurgicaux. Bien que la plupart des séries dans la littérature décrivent l’intérêt du traitement endoscopique et du shunt spléno-rénal proximal dans le traitement des varices gatsro-œsophagiennes et de l’hypertension portale [20-22], rares sont les séries qui développent la prise en charge de l’hypersplénisme et de la splénomégalie dans l’HTPNC [23]. Ohnishi et al a suggéré qu’à un stade précoce de la maladie, les veines portales peuvent être dilatées sans augmentation de la pression portale [24, 25]. Ceci est dû à une augmentation du flux sanguin portal par augmentation du flux sanguin de la veine splénique secondaire à la splénomégalie. On suggère que l’hypersplénisme est considéré comme l’un des facteurs d’une hypertension portale persistante même après l’éradication des varices œsophagiennes [26]. La splénectomie seule peut être justifiée dans la prise en charge de ce syndrome. Elle permet de traiter la splénomégalie et corriger l’hypersplénisme et par conséquent diminuer le débit sanguin portal. Mais elle peut conduire à une thrombose de la veine splénique qui pourrait être utilisée dans un éventuel shunt [27]. La ligature des varices oesophagiennes associé aux beta-bloquants est souvent indiquée pour traiter et prevenir l’hémorragie des varices oesophagiènnes comme dans le cas de l’HTP sur cirrhose [28, 29]. Dans une étude de Rajesh et al, parmi 55 patients opérés pour HTPNC, 2 patients ont bénéficié d’une splénectomie seule et chez 7 patients la splénectomie était associée au traitement endoscopique des varices oesophagiennes. Chez 2 malades parmi les 9 patients, l’évolution a été marquée par une récidive de l’hémorragie variqueuse [30]. Dans notre étude, tous nos malades ont bénéficié d’une splénectomie associée à une ligature des varices œsophagiennes avec un traitement d’entretien par les beta-bloquants. Les résultats étaient satisfaisants.

## Conclusion

Le syndrome de Banti doit être recherché systématiquement devant une hypertension portale avec absence d’une affection hépatique chronique. La splénectomie associée à la ligature des varices oesophagiènnes pourraient être suffisantes pour traiter ce syndrome et surtout ses conséquences sans avoir recours à une dérivation spléno-rénale.

### Etat des connaissances actuelles sur le sujet

- Le syndrome de Banti est une affection idiopathique;
- Le traitement consiste essentiellement sur les dérivations porto-systémiques.

### Contribution de notre étude à la connaissance

- Penser toujours à cette affection devant une splénomégalie avec foie sain;
- La splénectomie seule peut suffire pour traiter le syndrome de BANTI.

## Conflits d’intérêts

Les auteurs ne déclarent aucun conflit d’intérêt.
